# Oxygen Home

**Published:** 1897-07-10

**Authors:** 


### "OXYGEN HOME."

DR. GEORGE STOKER writes: I submit with great respect that the title "Oxygen Home" correctly describes the object and aims of that institution, as it was established to develop the system of treating wounds and ulcers by exposure to oxygen gas. My present view is that wounds (that have not healed by first intention) and ulcers should be treated in the way indicated, especially as by it a cicatrin is produced that closely resembles true skin. I can assure that it is not correct to state that "the cases in which oxygen alone is used did not do well, the cases of simple ulcers, wounds, burns, &c., have done well under this treatment. I have tried simple purified air, but without success, and this would seem to show that nitrogen has no effect and that it is only when additional oxygen is added that successful results are achieved.

*** As to our statement about the use of pure oxygen, which Dr. Stoker says is incorrect, we depended upon his own paper in the *British Medical Journal*, page 1,212, vol. ii., 1896, where he says, "It is found in practice that a solution as strong as 75 per cent. of pure oxygen, mixed with pure air, is too stimulating for any ordinary cases, and that with a weaker solution wounds heal better." We do not wish to say anything as to the success or otherwise of the treatment advocated; that is a matter which must be decided by experience. We think it a pity, however, that an institution should take to itself such a name as to indicate that it is confined to one single mode of treatment.—ED. *T. H.*

